# Heparan Sulfate: A Potential Candidate for the Development of Biomimetic Immunomodulatory Membranes

**DOI:** 10.3389/fbioe.2017.00054

**Published:** 2017-09-21

**Authors:** Bruna Corradetti, Francesca Taraballi, Ilaria Giretti, Guillermo Bauza, Rossella S. Pistillo, Federica Banche Niclot, Laura Pandolfi, Danilo Demarchi, Ennio Tasciotti

**Affiliations:** ^1^Department of Nanomedicine, Houston Methodist Research Institute, Houston, TX, United States; ^2^Department of Life and Environmental Sciences, Università Politecnica delle Marche, Ancona, Italy; ^3^Center for Biomimetic Medicine, Houston Methodist Research Institute, Houston, TX, United States; ^4^Department of Orthopaedic & Sports Medicine, The Houston Methodist Hospital, Houston, TX, United States; ^5^Center for NanoHealth, Swansea University Medical School, Swansea University Bay, Wales, United Kingdom; ^6^Department of Electronics and Telecommunications, Politecnico di Torino, Turin, Italy

**Keywords:** mesenchymal stem cells, microenvironment, immune response, regeneration, tissue engineering, biomolecules, heparan sulfate, collagen

## Abstract

Clinical trials have demonstrated that heparan sulfate (HS) could be used as a therapeutic agent for the treatment of inflammatory diseases. Its anti-inflammatory effect makes it suitable for the development of biomimetic innovative strategies aiming at modulating stem cells behavior toward a pro-regenerative phenotype in case of injury or inflammation. Here, we propose collagen type I meshes fabricated by solvent casting and further crosslinked with HS (HS-Col) to create a biomimetic environment resembling the extracellular matrix of soft tissue. HS-Col meshes were tested for their capability to provide physical support to stem cells’ growth, maintain their phenotypes and immunosuppressive potential following inflammation. HS-Col effect on stem cells was investigated in standard conditions as well as in an inflammatory environment recapitulated *in vitro* through a mix of pro-inflammatory cytokines (tumor necrosis factor-α and interferon-gamma; 20 ng/ml). A significant increase in the production of molecules associated with immunosuppression was demonstrated in response to the material and when cells were grown in presence of pro-inflammatory stimuli, compared to bare collagen membranes (Col), leading to a greater inhibitory potential when mesenchymal stem cells were exposed to stimulated peripheral blood mononuclear cells. Our data suggest that the presence of HS is able to activate the molecular machinery responsible for the release of anti-inflammatory cytokines, potentially leading to a faster resolution of inflammation.

## Introduction

Tissues consist of cellular and non-cellular components (Wagers, 2012; Perrini et al., 2016). The extracellular matrix (ECM) represents an intricate network of macromolecules, such as water, proteins, and polysaccharides that are produced by the cells and are assembled into an organized network in close association with the cell surface (Frantz et al., 2010). The ECM structure is often remodeled, and its molecular components are subjected to posttranslational modifications (Burghardt et al., 2002). The production and deposition of these components outside the cell surface provide structural and functional integrity to connective tissue and organs and act as physical scaffold for the cellular constituents. The role of ECM is also crucial in the response to growth factors, cytokines, and mechanical signals mediated by cell surface receptor, and in the provision of biochemical and biomechanical cues that are required for tissue morphogenesis, differentiation, and homeostasis (Schultz and Wysocki, 2009). Given the ability of ECM to provide biochemical and mechanical properties to any specific tissue (e.g., tensile and compressive strength and elasticity), its physical, topological, and biochemical composition is tissue specific, but largely heterogeneous, and depends on the biochemical and biophysical interaction of various cellular components (e.g., epithelial, fibroblast, adipocyte, and endothelial elements) and the cellular microenvironment (Brizzi et al., 2012). The relative amounts of the different types of matrix macromolecules and their organization in the ECM reflect the needs of the specific host tissue. Among all components of the ECM, glycosaminoglycans (GAGs) play a series of activities as they influence cell proliferation, differentiation, and gene expression (Oohira et al., 2000; Lamoureux et al., 2007), modulate protein gradient formation and signal transduction (Hacker et al., 2005). They are able to connect ECM structure, retain growth factors, maintain chemokines extracellular rate, and prevent growth factors degradation. In particular, heparan sulfate (HS) acts as cofactor in many biological processes, interacting with diverse protein ligands (such as growth factor, viral proteins, enzymes, protease inhibitors, and ECM molecules) (Perrimon and Bernfield, 2000; Dreyfuss et al., 2009). Furthermore, its activity changes if it is linked to the core protein or if it is free. It has been demonstrated that HS binds a wide variety of proteins (including chemokines, growth factors, morphogens, enzymes, and ECM component) that contain basic amino acids with a specific and direct interaction (Lortat-Jacob et al., 1995). HS activity influences cytokines processing and consequently their activity (Lortat-Jacob et al., 1995), while other ligands require some additional sequences to explicate their role, such as the interaction between HS and FGF receptor (Sasisekharan et al., 1997).

HS applied on mesenchymal stem cells (MSC) biology shows its ability to maintain cell growth, proliferation, viability, and stemness and decreasing the presence of apoptotic cells. Helledie et al. (2012) have recently evaluated the effect of HS on MSC immunophenotype, showing an increase in the expression of integrins (CD105 and CD73 molecules), a greater differentiative potential, and a more active secretion of molecules that support bone repair in an *in vivo* bone defect.

HS has been also demonstrated to play an important role in inflammation, due to its promising anti-inflammatory properties and the consequent potential to be used as therapeutic agent in some type of inflammatory diseases (Li and Vlodavsky, 2009). Such diseases include, for example, rheumatoid arthritis and diabetic nephropathy, where it helps prevent leukocyte adhesion and consequently the propagation of inflammation (Rops et al., 2004). Inflammation involves a series of events following a tissue injury caused by the presence of harmful stimuli, such as infection or physical damage. The first step of this reaction is the movement of T lymphocytes, monocytes, and neutrophils from the vascular system to damage site, with a cascade of biochemical reactions, which is activated to create the appropriate inflammatory response. These biochemical reactions increase the levels of chemoattractants, including complex components and cytokines, such as tumor necrosis factor-α (TNF-α) and interleukin-1β leading to an increased expression of the adhesion molecules (e.g., ICAM and VCAM) and selectins (Rops et al., 2004).

Therapeutically active MSC have been demonstrated to take part in the inflammatory cascade and contribute to tissue homeostasis by releasing trophic factors that act as anti-inflammatory immune modulators (Corradetti et al., 2012, 2014, 2016; Lange-Consiglio et al., 2016; Perrini et al., 2016). Their beneficial potential can be altered or improved through the exposure to bioactive molecules and the development of a naturally inspired bioactive material able to support and retain such capability in the context of injury or damage is still much needed (Willerth and Sakiyama-Elbert, 2008).

**Figure 1:**
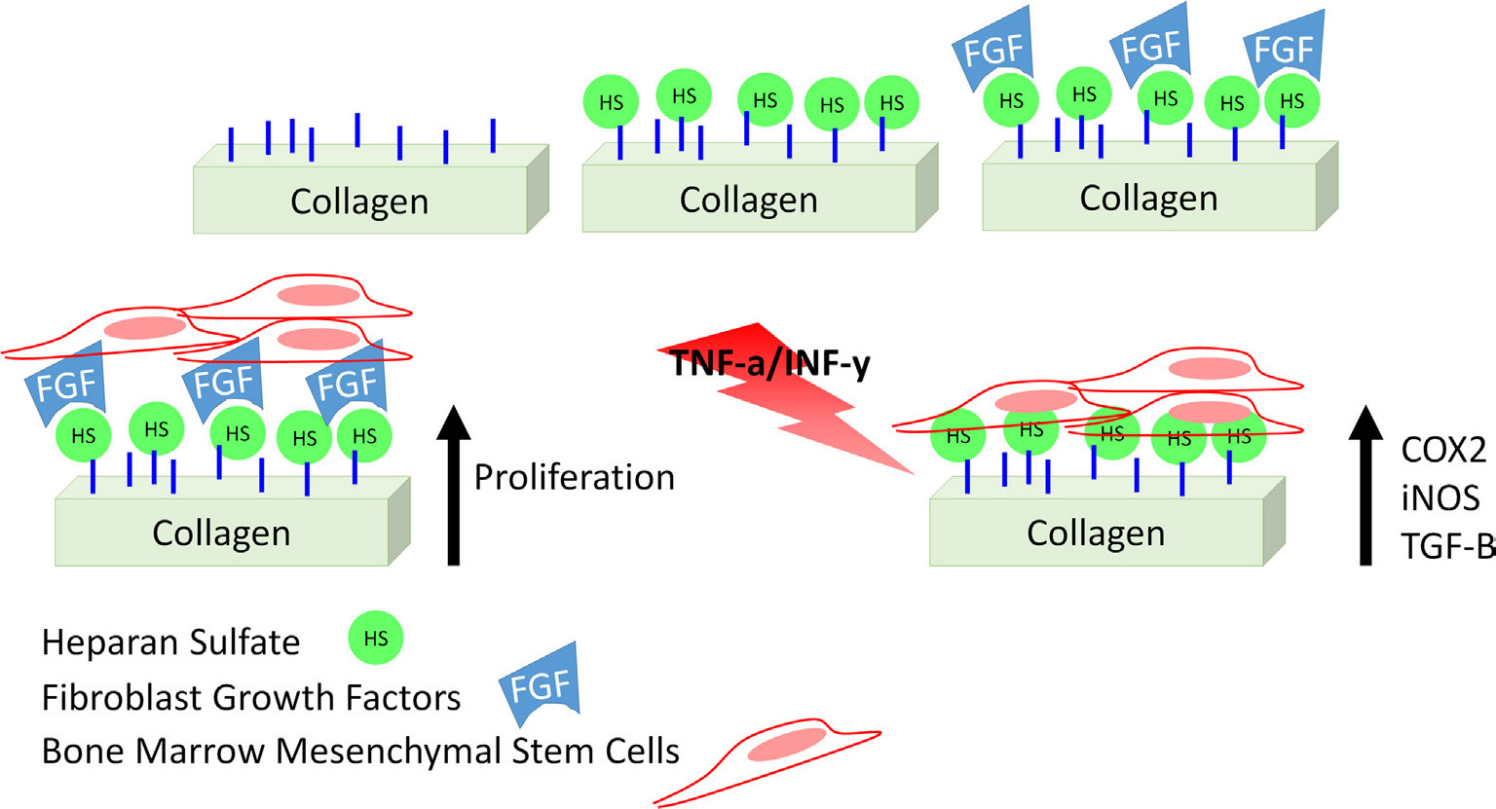
Schematic representation of the proposed study.

In this study, we describe a collagen-based mesh functionalized with HS with the aim of modulating inflammation by supporting MSC therapeutic potential. Although different studies describe a similar strategy (Meade et al., 2013; Mothéré et al., 2017), the translational point of view was never investigated, and the evaluation of the immunosuppressive potential of MSC on a bioactive material has never been provided. Figure 1 summarizes the approach we propose. In addition to describe the proposed material in terms of topography and stability of its functionalization, we evaluated the effect of such meshes to support the capability of MSC to adhere, proliferate, and maintain their conventional features, as well as to actively respond to an inflammatory environment, as we previously demonstrated in the context of cartilage-resembling materials (Corradetti et al., 2016; Taraballi et al., 2016). Using rat bone marrow-derived MSC as surrogate local progenitor cells, we investigated the influence of our biomimetic natural material in retaining the immunosuppressive potential of MSC either in standard conditions or in response to the combination of pro-inflammatory cytokines [TNF-α and interferon-gamma (IFN-γ)]. The capability of the proposed platform to support MSC anti-inflammatory role was finally proved in lymphocyte reaction assay.

## Materials and Methods

### Meshes Preparation and Characterization

Type I collagen mesh (Col) from bovine tendon (5% collagen gel in acetic acid) was produced by a solvent-casting method as previously described (Taraballi et al., 2013; Minardi et al., 2016c). Briefly, 1 g of type I collagen (Nitta Casings Inc.) was dissolved in an acetate buffer (pH 3.5) at the final concentration of 20 mg/ml. The collagen suspension was precipitated by the addition of sodium hydroxide (0.1 M) solution at pH 5.5. The resulting collagen slurry was mild crosslinked with 1.5 mM solution of 1,4-butanediol diglycidyl ether (Sigma-Aldrich) for 48 h. The crosslinked collagen was washed three times in water, and then the slurry was molden in metal racks, at a thickness of 2 mm and air dried under a fume hood for 3 days (final thickness: 0.1 mm). Covalent functionalization of heparan sulfate (HS) to collagen meshes was performed using the coupling reaction with 1-ethyl-3-(3-dimethyl aminopropyl)carbodiimide (EDC) and *N*-hydroxysuccinimide (NHS). After weight the collagen matrices, each sample was incubated in a solution of 50 mM 2-morpholinoethane sulfonic acid (pH 5.5) in ethanol. After 1 h, the samples were place in a solution of 5 mM EDC and 5 mM NHS and 0.2% (w/v) heparan sulfate (HS). After reaction for 24 h, the matrices were washed three times in PBS. For the stability study, collagen meshes have been incubated with the same concentration of HS in the absence of crosslinker.

#### Scanning Electron Microscopy (SEM) Imaging

HS-Col morphology was evaluated by SEM (Quanta 600 FEG, FEI Company, Hillsboro, OR, USA). Dried samples were sputter coated with 10 nm of Pt/Pd and imaged at a voltage of 10 mA.

#### Fourier Transform Infrared (FTIR)

Fourier transform infrared spectra were measured in attenuated total reflection using a Nicolet 6700 FT-IR Spectrometer (ThermoFisher Scientific). Col and HS-Col meshes were without any sample manipulation. Spectra were analyzed by the software EZ OMNIC (Nicolet) after baseline correction between 1,800 and 750 cm^−1^. The presence of HS was also confirmed by histochemical staining. The meshes were stained by Alcian Blue, which labels the proteoglycans.

#### Enzyme-Linked Lectin Assay (ELLA)

HS crosslinked and absorbed on collagen meshed has been incubate in PBS for 14 days at 37°C to evaluate the stability of binding between HS and collagen mesh. At each time, point the samples were treated with a solution of 2% BSA in PBS (100 µl) and shaken (14 h, 5°C). The patches after the blocking step have been incubated at room temperature with a solution of WGA peroxidase labeled (Sigma-Aldrich) (0.01 mg/ml, 200 µl) in PBS for 2 h with shaking. After three washes in PBS, we treated the samples with the peroxidase substrate OPD for 1 h. The absorbance was measured at 450 nm in triplicate.

### Cell Cultures

Rat compact bone MSC were isolated as previously reported (Corradetti et al., 2015) and maintained in standard culture media represented by Dulbecco’s Modified Eagle’s Medium (Sigma) supplemented with 10% fetal bovine serum (Euroclone), 1% l-glutammine (Gibco), and 1% antibiotic/antimycotic solution (containing penicillin, streptomycin, and amphotericin B, Gibco) at 37°C in a humidified atmosphere with 5% CO_2_. For maintenance of cultures, cells were plated at a cell density of 5 × 10^3^ cells/cm^2^. Medium was changed twice per week thereafter or according to the experiment requirements. Adherent cells were detached and subcultured using TrypLE Express (Invitrogen, ThermoFisher Scientific) before reaching confluence (80%) and subsequently replated at the same density for culture maintenance (Corradetti et al., 2016). When seeded onto meshes, MSC were harvested, resuspended in standard cell culture medium, and seeded at the density of 1 × 10^4^ cells/well in 12-well plates (Corning). Inserts (Corning) were used to hang membranes (Col and HS-Col) and allow for cell adherence onto the collagen without reaching well surface. Standard culture medium was then added to each well. Animal studies were conducted following approved protocols (AUP-0115-0002) established by Houston Methodist Research Institute’s Institutional Animal Care and Use Committee in accordance with the guidelines of the Animal Welfare Act and the Guide for the Care and Use of Laboratory Animals.

### MSC Characterization before Culture onto Meshes

Upon reaching confluence, cells were collected and characterized for the expression of MSC-associated markers (CD105, CD90, and CD105) with a FortessaTM cell analyzer (Becton Dickinson), under the assistance of the HMRI Flow Cytometry Core. Antibodies used (APC/Cy7-CD73, PE/Cy7-CD105, and Alexa Fluor 700-CD90) were purchased from BioLegend. Briefly, MSC were recovered by trypsin method and by spinning down at 500 *g* for 5 min. They were then washed with FACS buffer labeled with directly conjugated antibodies according to the manufacturer’s indications.

At passage 3, cells were also assessed for their capability to undergo osteogenic and chondrogenic differentiation following a previously reported procedure (Corradetti et al., 2015). Briefly, to induce osteogenesis, MSC were seeded at the density of 5,000 cells/cm^2^ in 12-well plates. Osteogenic induction was performed over 14-day period using a StemPro Osteogenesis Differentiation Kit (Gibco). To confirm differentiation, conventional von Kossa was performed. For chondrogenesis, cells were seeded at the density of 5,000 cells/cm^2^. Induction was performed using the StemPro Chondrogenesis Differentiation Kit (Gibco) for a 14-day period. ECM constituted by proteoglycans was visualized by conventional Alcian Blue staining.

### Effect of Fibroblast-Growth Factor (FGF) and HS on Cell Proliferation

To test the effect of HS in retaining fibroblast-growth factor (FGF) and improve cell proliferation, MSC were exposed to different conditions, and the growth rate recorded over a 7-day period was compared. Conditioned included the following: (1) MSC grown in 2D culture systems (CTRL) and treated with FGF (10 ng/ml, w/FGF; PeproTech), soluble HS (25 ng/ml, w/sHS; Carbosynth), and a combination of both (w/sHS and FGF), (2) MSC grown onto Col in presence of soluble HS (Col w/sHS), FGF (Col w/FGF), and a combination of the two (Col w/sHS and FGF), and (3) MSC grown onto HS-Col in presence in presence (HS-Col w/FGF) and absence (HS-Col) of FGF. Experiment was set in triplicate. MTT assay (Life Technologies) was performed according to the manufacturer’s procedures. The concentration of FGF to be used was previously defined in a range of 1–100 ng/ml (data not shown).

### Assessment of MSC Morphology, Metabolic Activity, and Immunofluorescence

Mesenchymal stem cells were seeded at the density of 100,000 cells/cm^2^ onto HS-Col, Col meshes and cultured for 24 h to assess their immediate response to the material in terms of proliferation, morphology, adherence, and MSC-associated genes expression. Cell proliferation onto membranes was evaluated by Alamar Blue (ThermoFisher Scientific) according to manufacturer’s instructions during a 3-day period as previously reported (Corradetti et al., 2016). Optical density was measured at wavelengths of 570 and 600 nm. Data are shown as mean of three independent biological replicates. Values have been reported as %AB over time, which is associated with the presence of metabolically active cells. For comparison, data obtained from MSC grown in two-dimensional conditions (CTRL) were also acquired.

Seven days after seeding, cellularized patches and 2D controls were washed and fixed in 4% PFA for 20 min. Cell membranes were then permeabilized with 0.1% Triton X-100 for 10 min and then blocked by 10% goat serum in PBS for 1 h at RT. Cells’ actin cytoskeletons were stained with phalloidin (Alexa Fluor 555, Invitrogen by Life Technologies) and DAPI to identify actin cytoskeleton at a dilution of 1/100 in blocking solution at RT in darkness, and subsequently the nuclei stained with DAPI (ThermoFisher Scientific Inc.) according to the manufacturer’s instructions. Samples were imaged with a confocal laser microscope (A1 Nikon Confocal Microscope), and the images were analyzed through the NIS-Elements software (Nikon). Col and HS-Col sample were imaged by confocal laser microscopy, and Z-stacks were acquired.

### Evaluation of the Immunosuppressive Potential of MSC Grown onto Meshes

To evaluate the efficacy of HS in supporting MSC retain their immunosuppressive potential, cells were seeded at the density of 100,000 cells/cm^2^ onto HS-Col, Col meshes and cultured for 24 h at 37°C before stimulation with pro-inflammatory cytokines occurred. Stimulation was performed as previously reported (Hegyi et al., 2012; Corradetti et al., 2016). Briefly, a combination of rat recombinant IFN-γ and TNF-α (20 ng/ml; PeproTech) was used and exposure lasted for 48 and 72 h. Cells cultured in 2D conditions (CTRL), whether stimulated (Positive CTRL) or not, were used as negative and positive controls, respectively. The expression of immunosuppressive markers was determined in also on MSC grown onto meshes in absence of stimulation. At each time point, the levels of expression of genes associated with MSC immunosuppressive potential were analyzed as follows.

### Peripheral Blood Mononuclear Cells (PBMC) Proliferation Test

Peripheral blood mononuclear cells were obtained from heparinized whole blood samples using density gradient centrifugation (Lymphoprep; Axis-Shield, Oslo, Norway) following the manufacturer’s instructions. PBMC proliferation was induced as previously reported (Corradetti et al., 2016). The effect of human MSC grown onto meshes (either Col or HS-Col) on T lymphocyte proliferation was determined after 48 h in a cell–cell contact setting with MSC and PBMC plated at a 1:10 ratio by using CellTrace CFSE Cell Proliferation Kit (Life Technologies). Briefly, 24 h after seeding the MSC onto meshes, PBMC were stained with CFSE following the manufacturer’s indications and cocultured. The percentage of proliferating PBMC in each experimental condition was analyzed by flow cytometry (Millipore).

### Gene Expression Analysis

Total RNA was extracted from cells using TRI Reagent^®^ (Sigma) and complementary DNA (cDNA) was synthetized from RNA using iScript™ cDNA Synthesis Kit (BioRad) according to the manufacturer’s protocol. Gene expression analysis was performed using specific oligonucleotide primers, designed based on rat (*Rattus norvegicus*) specific sequences and reported in Table 1. Pro-inflammatory markers include interleukin-6 (*Il-6*), *Tnf-α*, and inducible nitric oxide synthase (*iNos*). Immunosuppression-associated markers include prostaglandin E2 synthase (*Pges2*), transforming growth factor-beta (*Tgf-β*), and cyclooxygenase (*Ptgs2*). Mesenchymal-associated markers include *Cd90, Cd29*, and *Cd73*. Quantitative (real-time) PCR was executed using StepOne™ Real-Time PCR System with SYBR^®^ green method. The presence of pro-inflammation markers was tested on each sample, while immunosuppression-associated markers analysis was performed only on non-inflamed samples, at different growth conditions. Glyceraldehyde 3-phosphate dehydrogenase (*Gapdh*) was used as reference gene. Each sample was analyzed in triplicate.

**Table 1:** Oligonucleotide sequences used for quantitative RT-PCR analysis.

Gene	ID	Forward (5′–3′)	Reverse (5′–3′)	Length amplified (bp)
1. Interleukin-6 (*Il-6*)	NM_012589.2	CCTTCCCTACTTCACAAGTCC	ACAGTGCATCATCGCTGTTC	179
2. Tumor necrosis factor-α (*Tnf-α*)	NM_012675.3	GATCGGTCCCAACAAGGAGG	GCTTGGTGGTTTGCTACGAC	134
3. Nitric oxide synthase 2 (*iNos*)	NM_012611.3	GAGCATCCCAAGTACGAGTG	AATCTCGGTGCCCATGTACC	140
4. Prostaglandin E synthase (*Pge2*)	NM_021583.3	ATGAACCCACGCATTCGCTC	CTAGAAGGGGCACCAAGTCC	180
5. Transforming growth factor-beta (*Tgf-β*)	AY550025.1	AGGGCTACCATGCCAACTTC	CCACGTAGTAGACGATGGGC	167
6. Prostaglandin-endoperoxide synthase 2 (*Cox-2*)	NM_017232.3	CTCAGCCATGCAGCAAATCC	TCTTGTCAGAAACTCAGGCG	147
7. Thy-1 cell surface antigen (*Cd90*)	NM_012673.2	GACCCAGGACGGAGCTATTG	GCAGTCCAGTCGAAGGTTCT	138
8. Integrin subunit beta 1 (*Cd29*)	NM_017022.2	CGTGGTTGCCGGAATTGTTC	AGTTGTCACGGCACTCTTGT	163
9. 5′ Nucleotidase, ecto (*Cd73*)	NM_0215762	TCCTGCAAGTGGGTGGAATC	CCCCACCGTTGACCAGATAG	170

Differentiation assays were used to establish the plastic potential of the cells used in the study as well as to understand whether the structural and chemical stimuli provided by the meshes could induce cell reprogramming toward two lineages of the mesodermic layer. MSC grown in 2D and/or in 3D (Col or HS-Col) conditions were harvested following 14 days from induction or culture in standard media. RNA was extracted using RNeasy mini kit (Qiagen). cDNA was reverse transcribed from 150 ng of total RNA with iScript retrotranscription kit (BioRad Laboratories, Hercules, CA, USA). Amplifications were carried on using TaqMan^®^ Fast Advanced Master Mix on a StepOne Plus Real-Time PCR System (Applied Biosystems, Foster City, CA, USA). The following target probes (Applied Biosystems) were applied to evaluate the expression of: osteocalcin (*Bglap*) and alkaline phosphatase (*Alp*) for osteogenic differentiation. Transcription factors Sox-9 (*Sox9*) and Aggrecan (*Acan*) were evaluated for chondrogenic differentiation. The expression of each gene was first normalized to the level of housekeeping gene Beta Actin (*Actb*). Relative fold changes were normalized to the respective control. Technical triplicates for each biologic sample were evaluated, and results were reported as mean ± SD.

### Statistical Analysis

Statistical analysis was performed by using GraphPad Instat 3.00 for Windows (GraphPad Software, La Jolla, CA, USA, http://www.graphpad.com/). Three replicates to evaluate the metabolic state of the cells and gene expression were performed. The results are reported as mean ± SD, with *p* < 0.05 used as a threshold for significance. One-way analysis of variance for multiple comparisons by the Student–Newman–Keuls multiple comparison test was used.

## Results

### Material Characterization

**Figure 2:**
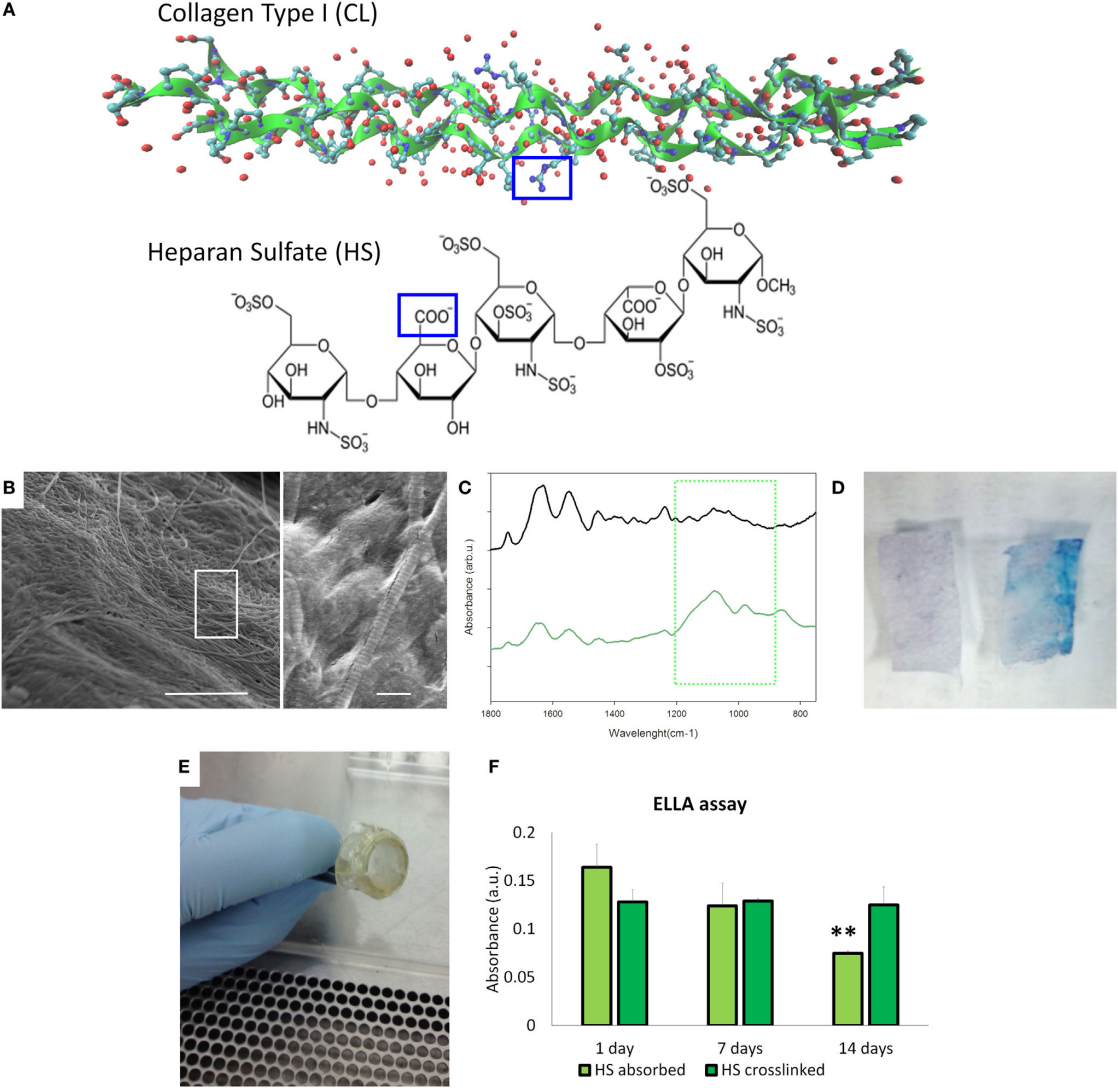
Material’s characterization. **(A)** Structural graft points on collagen ah heparan sulfate to be crosslinked by 1-ethyl-3-(3-dimethyl aminopropyl)carbodiimide/*N*-hydroxysuccinimide coupling. **(B)** Scanning electron microscopy images of HS-Col meshes (scale bar: 5 and 1 μm). **(C)** Fourier transform infrared/attenuated total reflection spectra of Col and HS-Col membrane. The green square highlighted the vibrational modes of polysaccharides of HS. **(D)** Alcian Blue staining of Col and HS-Col meshes. **(E)** Meshes assembled on the inserts to perform the following experiments. **(F)** Enzyme-linked lectin assay performed at 0, 7, and 14 days.

Collagen type I from bovine tendon was used for the preparation of meshes by the solvent-casting method. Since collagen contains several free amines groups (32/1,000 amino acid residues), we decided to use that as graph point for the dock of heparan sulfate (HS) that is characterized by the free carboxyl groups to obtain collagen type meshes functionalized with HS (HS-Col) (Figure 2A). The topography of the surfaces has been analyzed by SEM. Figure 2B shows the topography of the HS-Col demonstrating that the crosslink does not affect the collagen nanostructure. The presence of HS is confirmed by FTIR [Figure 2C shows the averaged (*n* = 3) of FTIR spectra of Col and HS-Col]. Amide I (1,700–1,600 cm^−1^) and Amide II (1,600–1,500 cm^−1^) are related to the stretching vibration of C=O bonds, and C–N stretching and N–H bending vibration respectively and are present in both samples, as well as the Amide III region (≈1,200–1,300 cm^−1^). In HS-Col, we found at ≈1,080 cm^−1^ (C–O stretching of carbohydrate residues in collagen and proteoglycans), ≈845 and ≈1,120 cm^−1^ (C–O–S stretching), and ≈1,397 cm^−1^ (COO– stretching of amino side chains) vibrational modes of GAGs (Corradetti et al., 2016). We also performed a simple Alcian Blue staining to confirm the presence of HS on the collagen surface (Figure 2D). After mounting the membrane on the insets (Figure 2E), we performed the ELLA. Although the presence of HS at the first time point was not significant different, the stability of the HS adsorbed is significantly lower, in fact after 14 days we lost the half concentration of HS onto the surface (Figure 2F).

### Effect of Fibroblast-Growth Factor (FGF) and HS on Cell Proliferation

**Figure 3:**
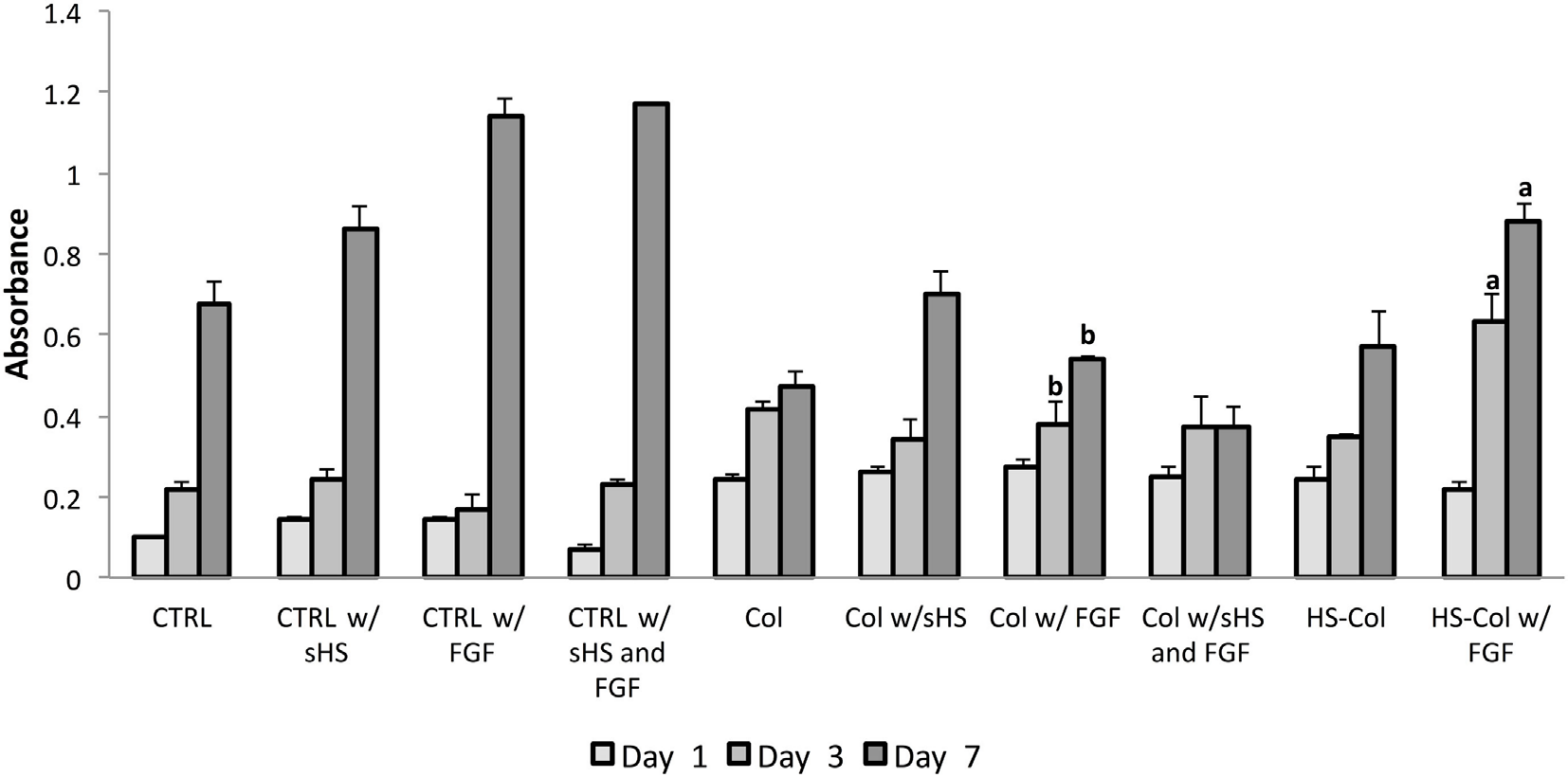
MTT assay results at various incubation times (1, 3, and 7 days) using rat mesenchymal stem cells cultured in different experimental conditions: in standard 2D culture (CTRL) with soluble HS (w/sHS) or soluble HS and Fibroblast growth factor (w/sHS and FGF), onto collagen-based membranes (Col), with soluble HS (Col w/sHS) or soluble HS and Fibroblast growth factor (Col w/sHS and FGF), and onto collagen-based membranes crosslinked with HS (HS-Col) and FGF (HS-Col w/FGF). Data are represented as mean of three independent biological replicates ± SD. Bars with different letters depict statistically significant differences (*p* < 0.01).

After ensuring the cell population was homogeneously positive for MSC-associated markers (Figure S1A in Supplementary Material) and confirming its differentiative potential toward osteogenic and chondrogenic lineages (Figure S1B in Supplementary Material), the efficacy of the proposed HS-Col meshes in acting as synthetic ECM and its capability to localize growth factors and improve MSC proliferation were assessed in the context of FGF over a 7-day period. MTT assay revealed that the presence of crosslinked HS allows for the fine control of FGF provided to cells (Figure 3). An overall decrease in cell proliferation was observed when cells were grown onto 3D structures, both Col and HS-Col compared to controls (CTRL), although the group HS-Col w/FGF showed a statistically significant (*p* < 0.01) increase compared to Col w/sHS and FGF at days 3 and 7. A block in cell growth was observed in the experimental group Col w/sHS and FGF, and no differences were shown between MSC grown in standard conditions supplemented with FGF (CTRL w/FGF) and a combination of FGF and sHS (CTRL w/sHS and FGF).

### Cell Viability, Morphology, and Spreading

**Figure 4:**
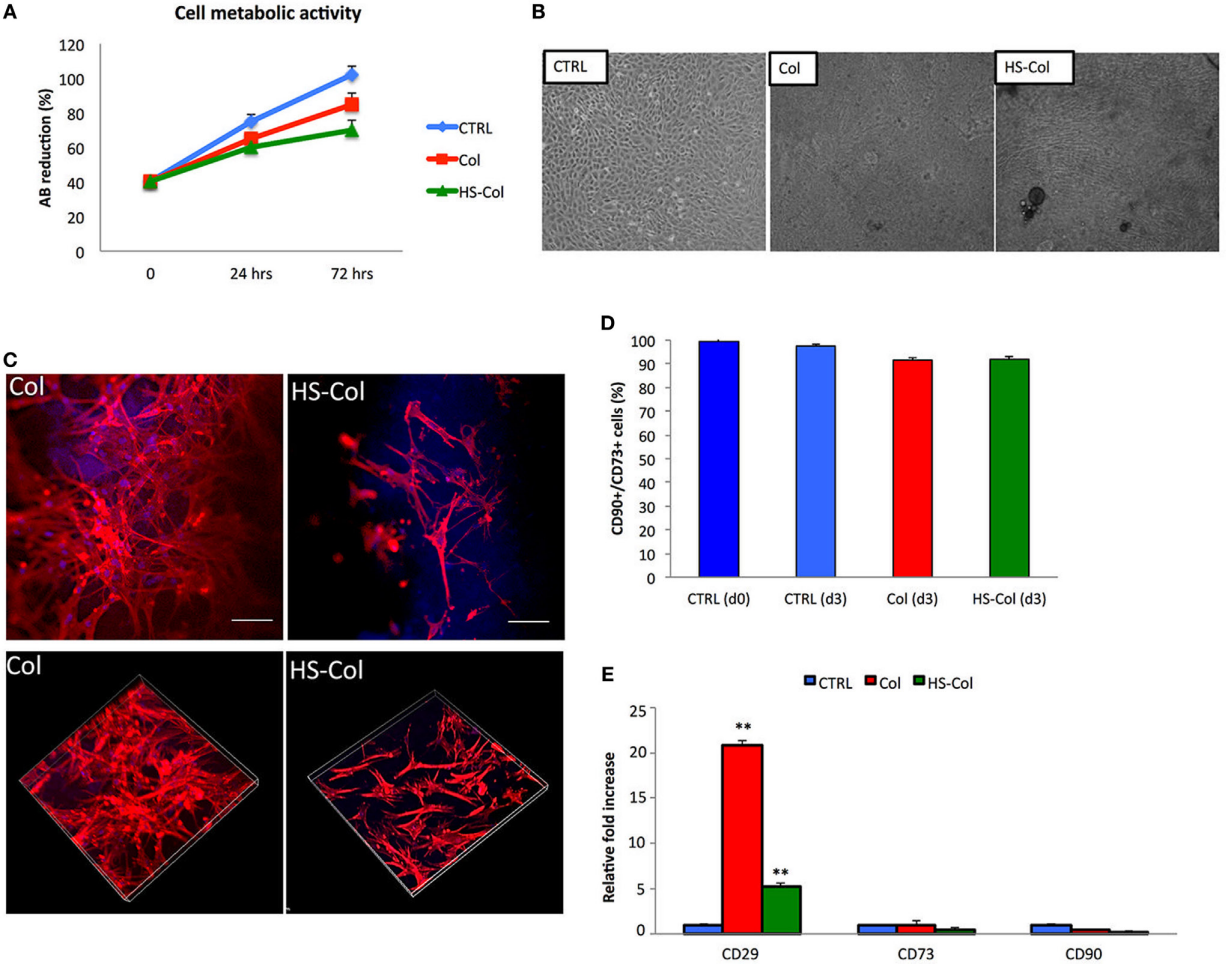
**(A)** Alamar Blue assay showing temporal changes (0, 24, and 72 h) in the %AB as representative of the presence of metabolically active cells in Col, HS-Col, and mesenchymal stem cells (MSC) grown in 2D conditions (CTRL). Data are shown as mean of three independent biological replicates ± SD. **(B)** Images representing cell morphology at 7 days onto Col, HS-Col, and in 2D conditions (CTRL). 10× magnification, scale bar: 20 µm. **(C)** Immunofluorescence staining with phalloidin (red), DAPI (blue), and vinculin (green) following 24 h culture onto Col and HS-Col. 20× magnification. Scale bars = 10 µm. **(D)** Histograms showing the percentage of CD90+/CD73+ cells at 3 days post HS-Col and Col-treatment. MSC grown in standard conditions a day 0 (CTRL d0) and day 3 (CTRL d3) are reported for comparison. **(E)** Comparison between MSC grown onto Col, HS-Col for the expression of MSC-associated markers (*CD29, CD73*, and *CD90*) at mRNA level at 24 h in culture. Data are represented as fold change compared with the expression levels found in cells grown in 2D conditions, CTRL (*n* = 3, **p < 0.01).

Alamar Blue assay demonstrated that both HS-Col and Col supported metabolically active cells growth during a period of 3 days (Figure 4A). As revealed by optical microscopy after 7 days of culture (Figure 4B), MSC spread across the meshes and reached confluence, as compared to 2D control (CTRL). Cells grown in presence of Col and HS-Col displayed a more spindle-shaped morphology compared to CTRL. To compare the cell structures formed by MSC adhering to meshes (both Col and HS-Col), antibodies to actin was used, and tridimensional images have been analyzed at confocal microscope (Figure 4C). Diffused cytoplasmic labeling was demonstrated when cells were seeded onto Col meshes, together with an intense staining confirming similar intracellular cytoskeleton organization. MSC grown onto HS-Col displayed a more elongated shape than their Col counterparts and maintained physical contact among them through extensions without completely spreading.

### MSC-Associated Markers Expression and the Differentiative Potential

Flow cytometry demonstrated a slight reduction in the percentage of MSC-associated markers at 3 days compared to controls (CTRL) following MSC culture onto HS-Col and Col (Figure 4D). To understand the effect of HS-Col on MSC at a genetic level, real-time PCR was used to evaluate the expression of mesenchymal-associated markers, including *Cd29, Cd73*, and *Cd90* (Figure 4E). Molecular analysis showed that at 72 h the expression of integrin *Cd29* was significantly (*p* < 0.01) higher in cells seeded onto Col than HS-Col and 2D controls (CTRL). A statistically significant increase compared to control (CTRL) in the expression levels of the same gene was found in HS-Col. The expression of *Cd73* and *Cd90* in cells treated with HS-Col was found lower than 2D control (0.475-fold and 0.155-fold, respectively). No significant differences were identified for the expression of these latter genes in Col.

Being cultured for 14 days onto meshes did not induce any increase in the expression of the osteogenesis-associated genes tested (Figure S2A in Supplementary Material), although the presence of collagen induced a statistically significant increase (*p* < 0.01) in the expression levels of the chondrogenic marker *Acan* compared to the 2D control. For this gene, values were assessed around 6.97 ± 1.71 and 10.43 ± 4.23 for Col and HS-Col, respectively. The presence of our meshes was associated with a great activation of both, osteogenic and chondrogenic genes, following 14 days of induction toward the two lineages (Figures S2B and S3 in Supplementary Material).

### Immunosuppressive Potential

**Figure 5:**
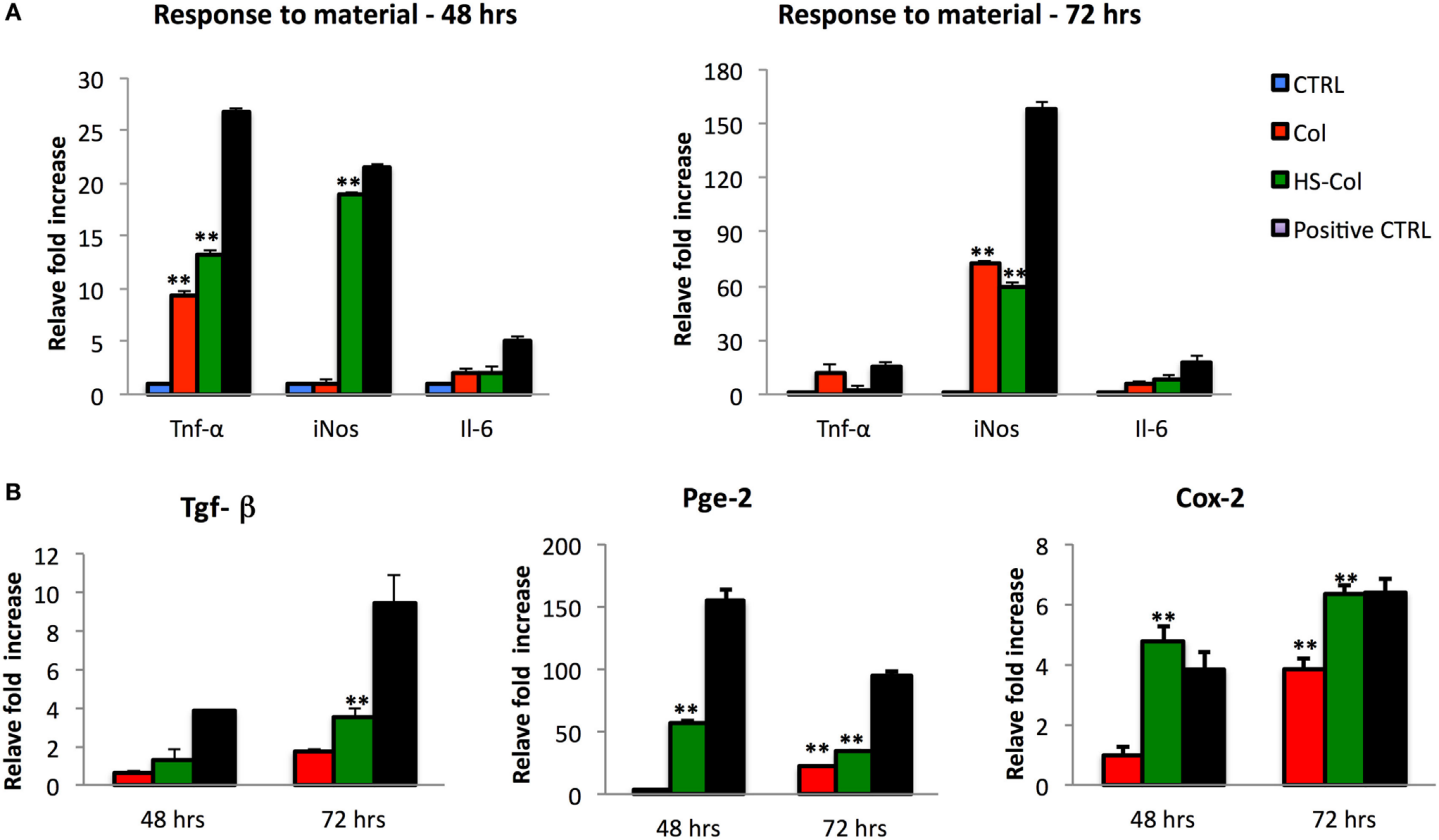
Comparison between mesenchymal stem cells grown onto Col and HS-Col for the expression of pro-inflammatory (*Tnf-α, iNos*, and *Il-6*) **(A)** and anti-inflammatory (*Tgf-β, Pge2*, and *Cox-2*) **(B)** markers at mRNA level after 48 and 72 h. Data are represented as fold change compared with the expression levels found in cells grown in 2D conditions, CTRL. Cells treated with a mix of pro-inflammatory cytokines [interferon-gamma and tumor necrosis factor-α (TNF-α and IFN-*γ*, 20 ng/ml)] were used as positive control (Positive CTRL) (*n* = 3, **p < 0.01).

To understand whether the exposure of MSC to HS-Col and Col could represent a source of stress for the cells, their response to the material was tested in absence of any other pro-inflammatory molecule. At 48 and 72 h, the levels of expression of genes encoding for immunosuppressive factors (*Tnf-*α, *iNos*, and *Il-6*) were evaluated in comparison with untreated cells (CTRL) or inflamed cells used as positive controls. Figure 5A shows a great increase in the expression of *Tnf-α* and *iNos* in cells grown onto HS-Col for 48 h compared to CTRL (13.32 ± 0.23 and 19.02 ± 0.15, respectively). No significant differences in the expression levels of *Il-6* were found in Col and HS-Col at the same time point or at 72 h. Interestingly, the expression of *Tnf-α* diminished overtime in presence of HS, showing lower values than Col (2.11 ± 0.11 vs 12.24 ± 1.3). The *iNos* expression was found even increased at 72 h in both Col and HS-Col. Concomitantly, a significant increase in the expression of markers mediating the reduction of inflammation (*Tgf-β, Pges2*, and *Ptgs2*) was observed at 48 h in comparison with the CTRL and Col, with values assessed around 3.83 ± 0.50 for *Tgf-β*, 4.78 ± 0.50 for *Ptgs2*, and 57.41 ± 8.60 for *Pges2* (Figure 5B). Values were found even increased at 72 h in the case of *Ptgs2* and *Tgf-β* (6.35 ± 0.29 and 9.46 ± 0.38), the same trend being observed also for cells exposed to Col. The expression levels of *Pge2* were found reduced overtime when cells were grown onto HS-Col.

**Figure 6:**
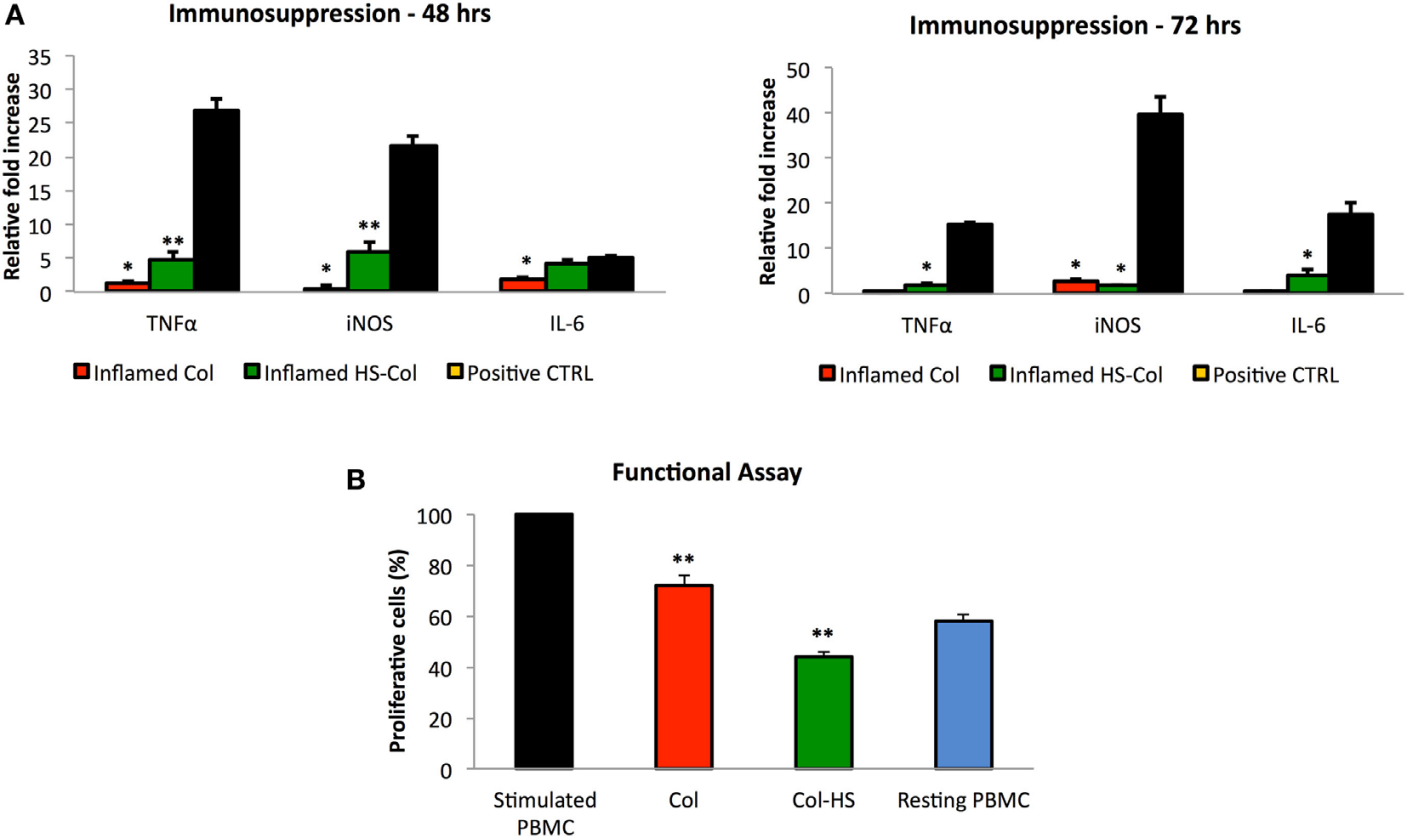
Expression at mRNA level of immunosuppression-associated genes (*Tnf-α, iNos*, and *Il-6*) of mesenchymal stem cells (MSC) grown onto Col (Inflamed Col) and HS-Col (Inflamed HS-Col) following 48 and 72 h stimulation with pro-inflammatory cytokines [interferon-gamma (IFN-γ) and tumor necrosis factor-α (TNF-α), 20 ng/ml] **(A)**. Data were normalized to the respective untreated control (CTRL, value = 1) (*n* = 3; **p < 0.01). Cells treated with a mix of pro-inflammatory cytokines (IFN-γ and TNF-α, 20 ng/ml) were used as positive control (Positive CTRL). Effect of HS-Col on the proliferation of stimulated peripheral blood mononuclear cells (PBMC) after 48 h of coculture with MSC grown onto meshes **(B)**. For comparison, the percentage of stimulated PBMC and resting PBMC is also reported. Data are represented as the mean of three biological replicates ± SD. Asterisks depict highly significant (**p < 0.01) and significant (*p < 0.05) differences compared with stimulated PBMC.

To test the efficacy of the proposed material to support MSC immunosuppressive potential, cells grown onto Col and HS-Col meshes were stimulated with the pro-inflammatory cytokines TNF-α and IFN-γ (at the concentration of 20 ng/ml) for 48 and 72 h. qPCR was used to evaluate the relative amount of genes responsible for the production of pro-inflammatory molecules, which are responsible for the activation of such process, including *Tnf-*α, *iNos*, and *Il-6*. Data were normalized to the respective untreated control (CTRL, value = 1). Consistent with the previous findings, in response to stimulation cells grown onto Col produced nearly identical amounts of immunosuppressive genes (*iNos* and *Tnf-α*) compared with those cultured in 2D and standard conditions, whereas cells grown onto HS-Col scaffolds produced significantly higher amounts. Figure 6A shows a significant increase in the expression of *Tnf-α, iNos*, and *Il-6* in MSC grown onto inflamed HS-Col compared to those seeded onto inflamed Col at 48 h. Values were assessed around 4.82 ± 1.23, 6.03 ± 1.46, and 4.24 ± 0.41 for *Tnf-a, iNos*, and *Il-6*, respectively. At 72 h, a marked decrease in the expression of *iNos* was found in inflamed HS-Col compared to 48 h (6.03 ± 1.46 vs 1.88 ± 0.22), which was found even lower than the expression levels found in Col at the same time point (2.63 ± 0.52). Finally, when the immunosuppressive potential of MSC grown onto HS-Col was evaluated in a lymphocyte reaction assay, a marked reduction in the proliferation of PHA-stimulated PBMC was noticed after 2 days of coculture compared to their Col counterparts, with percentage of proliferative cells assessed around 44 and 72%, respectively (Figure 6B).

## Discussion

Stem cells are widely used in regenerative medicine applications because of their properties, particularly their ability to secrete bioactive molecules that are known to have an immunosuppressive potential and to participate in the recreation of the correct pro-regenerative microenvironment following an injury (Caplan, 2007). They are closely associated with surrounding environment, which is responsible for influencing the way they respond to stimuli. The ability of biomaterials mimicking the ECM to promote constructive tissue remodeling can be attributed to both their structure and composition. Several tissue engineering-based approaches have been developed so far in the attempt to recapitulate the composition of the ECM (e.g., collagen and GAGs) and provide endogenous cells with the physical, chemical, and mechanical stimuli needed to support their biological activities during development, homeostasis, and response to injury (Corradetti, 2017). We recently developed collagen-based scaffolds functionalized with chondroitin sulfate and demonstrated their capability to induce a cartilage-resembling environment able to support stem cell differentiation and immunosuppression, thus holding the promise for the treatment of orthopedic chronic diseases (Corradetti et al., 2015, 2016; Fernandez-Moure et al., 2015; Minardi et al., 2015, 2016a,b; Perrini et al., 2016; Taraballi et al., 2016). Among GAGs, also HS has been proposed to take part in ECM composition, to retain growth factors and chemokines and prevent growth factors degradation (Dreyfuss et al., 2009). In the process of developing our biomimetic materials for tissue-engineering applications, we first tried to understand how HS bioactivity could change when provided as soluble molecule, or absorbed/crosslinked onto collagen-based membranes. We confirmed that HS influences the activity of FGF and actively participates in the presentation of the ligand on the receptor improving its activation and consequently cell proliferation (Lortat-Jacob et al., 1995; Sasisekharan et al., 1997; Dreyfuss et al., 2009). We also provided evidences about the fact that HS supports such activity when crosslinked onto collagen-based scaffolds and it is significantly reduced when it is in its soluble form or absorbed.

We then proposed collagen-based membranes crosslinked with heparan sulfate, which mimics the ECM of connective tissues to study their effect on MSC proliferation, adhesion, gene expression, as well as on their potential to release immunosuppressive molecules suggested to lead to the resolution of inflammation following an injury or an implant.

Biomimetic meshes could be very usefully for the repair of different tissues. We recently demonstrated that could be a valid alternative to the clinical polymeric materials used in soft tissue repair (Minardi et al., 2016c) as well as in cell delivery for more regenerative medicine approaches (Pandolfi et al., 2017).

In agreement with the existing literature and with our previous observations (Taraballi et al., 2014, 2016; Minardi et al., 2016a,b), data presented herein show that in presence of a 3D culture system cells proliferation appears to be slower compared to cells cultured in two-dimensional standard conditions, because of the different growth surface and the need of differently reorganizing. This statement supports the apparent discrepancies between the metabolic state of the cells and their proliferative potential. Our findings are confirmed by the ICC analysis revealing that cells grown onto membranes tend to form focal adhesion and to create a bond between the synthetic ECM and their cytoskeleton (Wozniak et al., 2004). To compare the cell adhesion structures formed by MSC adhering to the two matrices tested (Col and HS-Col), the presence of different phenotype, which are presumably activated through the interaction between integrin receptors on cells, was further confirmed by the high expression of *CD29* (or integrin-β1) at a molecular level, 72 h after seeding. Our data suggest that MSC are able to create a strong connection with the collagen substrate, being the most represented protein in ECM (Hynes and Naba, 2012).

These observations were further confirmed by the levels of *Cd73* and *Cd90* expression, as demonstrated by real-time PCR. Their expression levels in cells grown within HS-Col were found lower than control. Especially, *Cd90* appears to be significantly down-regulated respect of control. According to these results, Ode et al. (2013) have proved that a downregulation of *Cd73* expression is observed during differentiation, particularly during chondrogenesis, and when cells are exposed to mechanical stimulation. In other studies it has also been demonstrated that a decreasing ability of cells to migrate is associated with a lower expression of *Cd73* and *Cd29*, and the reduction of mRNA level of *Cd90* after mechanical stimulation, as well as during the differentiation (Wiesmann et al., 2006; Ode et al., 2011). Based on this, we can speculate that membranes cause a sort of stress in cells thus inducing a down regulation of the typical mesenchymal-associated markers. If this is the case, MSC grown onto HS-Col membranes lose their undifferentiated state and the stress they undergo could eventually lead their specification toward a more committed lineages (Helledie et al., 2012), as suggested by the matrix deposition revealed in Figure 4B. We next assessed the immunosuppressive potential of these membranes and, at the same time, their ability to support the immune-regulatory capability attributed to MSC (Caplan and Sorrell, 2015) in response to pro-inflammatory stimuli for their potential use in regenerative medicine applications. The first step was to evaluate the expression of pro-inflammatory molecules (*Tnf-α, iNos*, and *Il-6*) at mRNA level, following 48 and 72 h culture onto Col or HS-Col, in the attempt to understand whether or not the contact with collagen or HS-Col and their topographical properties could affect stem cell immunosuppressive behavior. Our data demonstrated that high levels of the iNos were produced by cells exposed to HS-Col compared to cells grown onto Col or in 2D conditions, with values that were found reduced compared to other experimental groups at 72 h. To date, these molecules are released by MSC to contrast or modulate the activities of several immune cell types, included but not limited to T and NK cells proliferation, B cell maturation, macrophages polarization, and dendritic cell maturation activation. This suggests that the 3D environment provided by HS-Col resulted in a greater stress for cells, probably due to the different mechanical stimuli that the material induces in the cells. Interestingly, however, such activity was further stimulated when cells were inflamed with TNF-α (20 ng/ml) and IFN-γ (20 ng/ml), leading to a marked accumulation (*p* < 0.01) of pro-inflammatory molecules, as observed in HS-Col compared to Col. These data were corroborated by the significant increase in the expression of genes encoding for other immunosuppressive molecules, which are normally associated with the resolution of inflammation, such as *Pges2, Ptgs2*, and *Tgf-β* (Zinocker and Vaage, 2012; Zinocker et al., 2012). To further validate the potential of HS-Col meshes to support MSC immunosuppression and understand whether the bioactivity of the immune modulating molecules produced by MSC following stimulation could lead to a reduced T cell proliferation (Zinocker and Vaage, 2012; Zinocker et al., 2012), the effect of HS-Col was tested in a functional assay. A greater capability to inhibit T cell growth was detected when MSC were grown onto meshes (both Col and HS-Col) although the presence of HS crosslinked onto the collagen membranes was associated with a greater MSC immunogenic capability (Corradetti et al., 2016).

## Conclusion

Here, we proposed HS-functionalized biomimetic meshes resembling the composition of ECM of soft tissue. We demonstrated that the surface modifications applied on the collagen-based material affect MSC behavior and improve their immunosuppressive competence. The proposed material represents a proof of concept for further developing immunomodulatory materials with the capability of supporting and enhancing MSC potential, and ultimately reducing inflammation at the site of implant.

## Author Contributions

BC and FT conceived the idea for this project. BC and FT designed and conducted the experiments. BC and FT wrote the manuscript. FT prepared meshes and performed their chemical characterization, cells characterization and staining assisted by GB, FBN and LP. BC conducted the cellular and molecular work assisted by IG, GB and RSP. DD and ET provided mentoring and contributed the funding support.

## Conflict of Interest Statement

The authors declare that the research was conducted in the absence of any commercial or financial relationships that could be construed as a potential conflict of interest.

## Supplementary Material

Figure S1 Mesenchymal stem cells (MSC) characterization. **(A)** Flow cytometric analysis showing MSC are homogeneously positive for CD90, CD73, and CD105. **(B)** Differentiative assay performed on MSC demonstrated they are prone to undergo osteogenic and chondrogenic specification. The expression of osteoblastogenesis (*Alp* and *Bglap*)- and chondrogenesis (*Acan* and *Sox9*)-associated markers was quantified by real-time PCR. Data were normalized to the value obtained from uninduced cells (CTRL, value = 1) (*n* = 3).

Figure S2 **(A)** Effect of HS-Col meshes on the differentiative potential of mesenchymal stem cells. Cells grown onto Col and HS-Col were evaluated for the expression of osteoblastogenesis (*Alp* and *Bglap*)- and chondrogenesis (*Acan* and *Sox9*)-associated markers at 14 days. Data were normalized to the value obtained from cells grown in 2D conditions (CTRL, value = 1) (*n* = 3). **(B)** Analysis of the same genes following 14 days of induction. Data were normalized to the values obtained from the respective uninduced cells (*n* = 3).

Figure S3 Differentiative assays showing the capability of mesenchymal stem cells (MSC) to differentiate toward the osteogenic and chondrogenic lineages following 14 days of induction. von Kossa and Alamar Blue stainings were used to highlight matrix deposition in MSC grown in standard conditions (CTRL) or onto Collagen (Col) and heparan sulfate (HS)-functionalized Collagen (HS-Col) meshes. Scale bars: 20 μm.
