# Corrigendum: Heparan Sulfate: A Potential Candidate for the Development of Biomimetic Immunomodulatory Membranes

**DOI:** 10.3389/fbioe.2017.00086

**Published:** 2018-01-29

**Authors:** Bruna Corradetti, Francesca Taraballi, Ilaria Giretti, Guillermo Bauza, Rossella S. Pistillo, Federica Banche Niclot, Laura Pandolfi, Danilo Demarchi, Ennio Tasciotti

**Affiliations:** ^1^Department of Nanomedicine, Houston Methodist Research Institute, Houston, TX, United States; ^2^Department of Life and Environmental Sciences, Università Politecnica delle Marche, Ancona, Italy; ^3^Center for Biomimetic Medicine, Houston Methodist Research Institute, Houston, TX, United States; ^4^Department of Orthopaedic & Sports Medicine, The Houston Methodist Hospital, Houston, TX, United States; ^5^Center for NanoHealth, Swansea University Medical School, Swansea University Bay, Wales, United Kingdom; ^6^Department of Electronics and Telecommunications, Politecnico di Torino, Turin, Italy

**Keywords:** mesenchymal stem cells, microenvironment, immune response, regeneration, tissue engineering, biomolecules, heparan sulfate, collagen

## Additional Affiliation(s)

In the published article, there was an error regarding the affiliation **[6]** for **Federica Banche Niclot**. As well as having affiliation **[3]**, they should also have **[6]**. The authors apologize for this error and state that this does not change the scientific conclusions of the article in any way.

## Addition of an Author

**[Danilo Demarchi]** was not included as an author in the published article. The authors apologize for this error and state that this does not change the scientific conclusions of the article in any way.

## Missing Citation

In the original article, **Corradetti et al. (2012)** was not cited in the article. The citation has now been inserted in **[Introduction]** and should read:

**Therapeutically active MSC have been demonstrated to take part in the inflammatory cascade and contribute to tissue homeostasis by releasing trophic factors that act as anti-inflammatory immune modulators (Corradetti** et al.**, 2012, 2014, 2016; Lange-Consiglio** et al.**, 2016; Perrini** et al.**, 2016). Their beneficial potential can be altered or improved through the exposure to bioactive molecules and the development of a naturally inspired bioactive material able to support and retain such capability in the context of injury or damage is still much needed (Willerth and Sakiyama-Elbert, 2008).**

The authors apologize for these errors and state that this does not change the scientific conclusions of the article in any way. The original article has been updated.

## Author Contributions

BC and FT conceived the idea for this project. BC and FT designed and conducted the experiments. BC and FT wrote the manuscript. FT prepared meshes and performed their chemical characterization, cells characterization and staining assisted by GB, FBN and LP. BC conducted the cellular and molecular work assisted by IG, GB and RSP. DD and ET provided mentoring and contributed the funding support.

## Conflict of Interest Statement

The authors declare that the research was conducted in the absence of any commercial or financial relationships that could be construed as a potential conflict of interest.
