# Supplementary figures and images for: Heparan Sulfate: A Potential Candidate for the Development of Biomimetic Immunomodulatory Membranes
Source: Front Bioeng Biotechnol. 2017 Sep 21;5:54. doi: 10.3389/fbioe.2017.00054 (PMC5613095; doi:10.3389/fbioe.2017.00054)

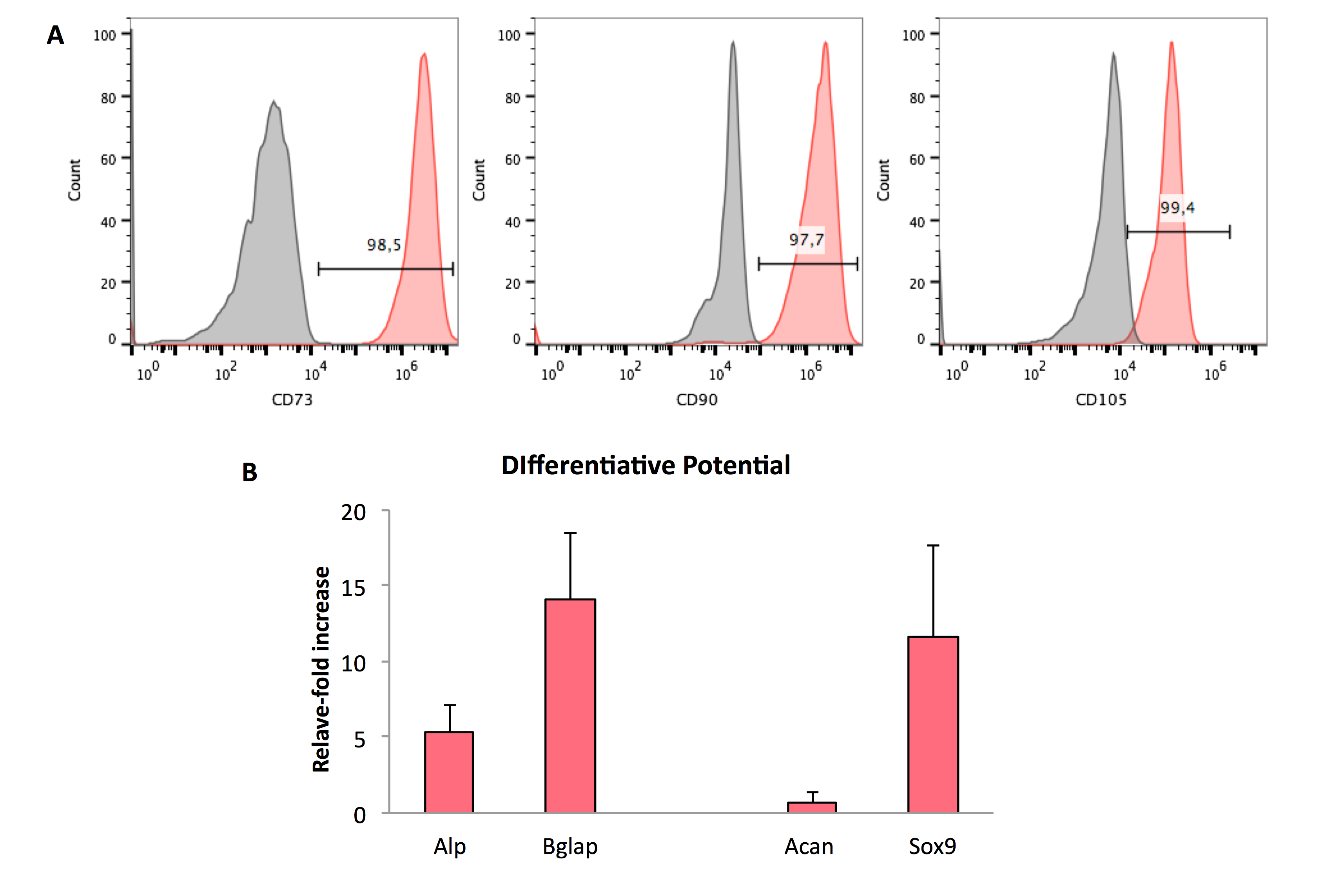

Supplement: Figure S1 — Mesenchymal stem cells (MSC) characterization. (A) Flow cytometric analysis showing MSC are homogeneously positive for CD90, CD73, and CD105. (B) Differentiative assay performed on MSC demonstrated they are prone to undergo osteogenic and chondrogenic specification. The expression of osteoblastogenesis (Alp and Bglap)- and chondrogenesis (Acan and Sox9)-associated markers was quantified by real-time PCR. Data were normalized to the value obtained from uninduced cells (CTRL, value = 1) (n = 3). [file Image_1.TIF]

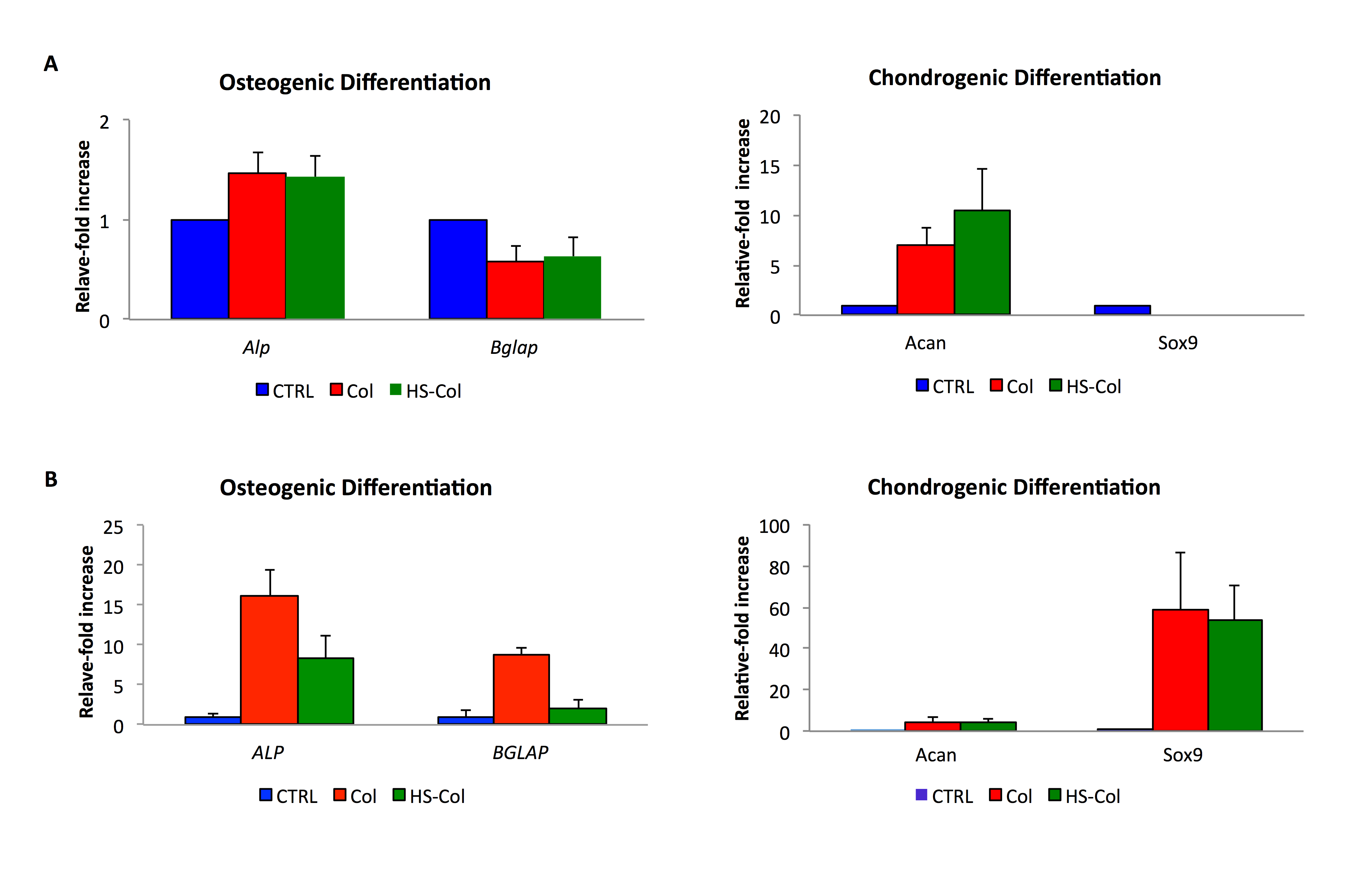

Supplement: Figure S2 — (A) Effect of HS-Col meshes on the differentiative potential of mesenchymal stem cells. Cells grown onto Col and HS-Col were evaluated for the expression of osteoblastogenesis (Alp and Bglap)- and chondrogenesis (Acan and Sox9)-associated markers at 14 days. Data were normalized to the value obtained from cells grown in 2D conditions (CTRL, value = 1) (n = 3). (B) Analysis of the same genes following 14 days of induction. Data were normalized to the values obtained from the respective uninduced cells (n = 3). [file Image_2.TIFF]

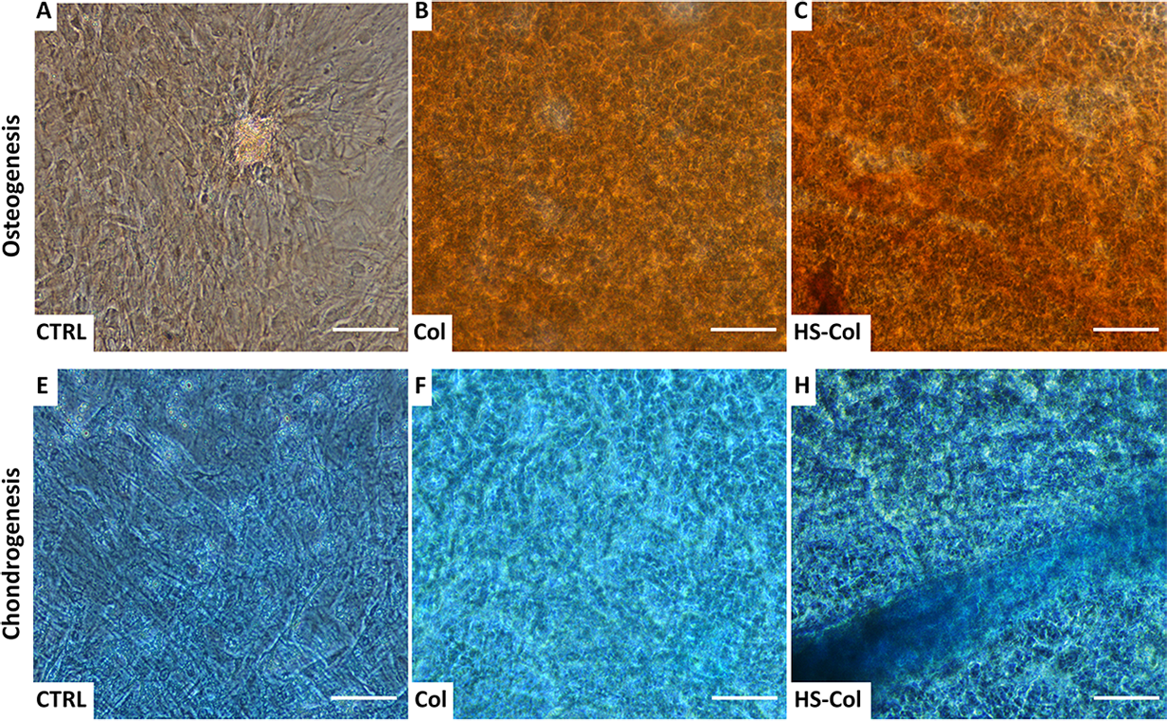

Supplement: Figure S3 — Differentiative assays showing the capability of mesenchymal stem cells (MSC) to differentiate toward the osteogenic and chondrogenic lineages following 14 days of induction. von Kossa and Alamar Blue stainings were used to highlight matrix deposition in MSC grown in standard conditions (CTRL) or onto Collagen (Col) and heparan sulfate (HS)-functionalized Collagen (HS-Col) meshes. Scale bars: 20 μm. [file Image_3.TIF]
